# Computational Biology in Brazil

**DOI:** 10.1371/journal.pcbi.0030185

**Published:** 2007-10-26

**Authors:** Goran Neshich

**Affiliations:** University of California San Diego, United States of America

## Introduction

At the request of the *PLoS Computational Biology* Editor-in-Chief, I agreed to write about computational biology in Brazil (see author information in Box 1). That meant describing: a) the history of the field in our country (short as the history of the field itself is short); b) the current state of the field in Brazil; c) the influence of computational biology–related technologies on the development of the national economy; d) the entrepreneurship that is rising from already-established academic activities; and e) educational activities ongoing or planned which are deemed necessary to establish the required critical mass of well-trained specialists. Why is an article like this important now? It is estimated that Brazil combined with China, Russia, and India will have a larger gross national product (GNP) than the US, Japan, Germany, and the UK combined by 2020. In short, we can expect Brazil to have significant impact on the field of computational biology in the years to come, and now is the time to explore that promise.

## Increased Economic Activity Boosted Research Investment

Brazil is a country with almost 190 million inhabitants that occupies an area equal to 81% of the area of Europe and 115% of the area of the US, excluding Alaska and Hawaii. In the late 1980s, Brazil left behind its mantle as a simple coffee producer with an agricultural boom, offering the world market major commodities such as soybeans, meat, poultry, and, more recently, fruit and flour. The rise in agricultural production came via a well-constructed socioeconomic plan put together by the Brazilian government, including targeted investment in supporting science and technology. As a consequence, Brazil became, for example, one of the major exporters of medium-sized aircraft. Further, the Brazilian petroleum company, Petrobras, provides Brazil self-sufficiency in oil, and is a major catalyst for the widespread use of ethanol in automobiles. The increasing economic activity enabled the country to invest proportionally in its scientific research.

## Government Policies for Research and Development

As part of this investment, the Brazilian government showed specific interest in biotechnology, and I would argue that the current favorable situation, in which computational biologists find themselves, has deep roots in the long-term dedication of policymakers to biotechnology.

In the 1920s and 1930s the first Brazilian universities were established. The largest of them all, USP (University of São Paulo), was started in 1934. Today, USP is responsible for more than a quarter of the total publications in Brazil in almost every scientific area, even though there are more than 100 universities in Brazil now. In terms of productivity, three other universities closely follow: UNICAMP in Campinas, in the state of São Paulo, UFRJ in Rio de Janeiro, and UFMG in Belo Horizonte. Notably, these four universities together with EMBRAPA (Brazilian enterprise for research in agriculture, a close equivalent to the US Department of Agriculture), Fiocruz, and Ludwig Institute, both of which are involved in human health related research, and the National Laboratory for Scientific Computation (LNCC), are responsible for most of the scientific publications in computational biology.

To consolidate its efforts in building consistent policies for scientific growth and to create a critical mass of specialists in various areas of science, in 1951 the Brazilian government established the National Research Council (CNPq), and the National Agency for Scholarly Advancement in Scientific Research (CAPES). The former agency was focused on guiding national scientific advancement through financing research projects and collaborations, while the latter provided scholarships to anyone who wanted to obtain higher degrees (Master's, Ph.D.) and to undertake postdoctoral training, either here in Brazil or abroad. In parallel, Brazilian states initiated their own Scientific Research Agencies (FAP) and São Paulo started with its own, FAPESP, in 1958. As São Paulo is the richest state in the Brazilian federation, the impact of FAPESP investments on science and technology in that particular region were, and are, substantial. The positive impact of FAPESP can be attributed to at least two factors. First, São Paulo has a law stating that 1% of the tax revenue collected by the state has to be given to FAPESP, and, second, the administrative costs of FAPESP are limited to not more than 5% of its total budget.

On the national level, in 1981 the Brazilian government initiated a broad program (PRONAB) for supporting scientific research in biotechnology. A further government program, PADCT, was initiated in 1984. As a consequence of these national programs, Brazil has established a critical mass of well-trained specialists and equipped many laboratories, which were fully functional by the early 1990s. These programs notwithstanding, Brazil remains below many countries with only 12% of 17- to 25-year-olds studying at universities compared with approximately 50% in the United States.

**Box 1. Author Biography**

**Goran Neshich, Ph.D.,** is the Structural Computational Biology (SCB) group leader at the Brazilian Agricultural Research Corporation (EMBRAPA), National Agricultural Information Technology Research Center (CNPTIA), Campinas, São Paulo, Brazil, and associate professor at UNICAMP's Department of Biology. After obtaining his Ph.D. in molecular biophysics from the University of Illinois Urbana-Champaign (Don DeVault's laboratory) in 1989, Neshich conducted his postdoctoral research with Barry Honig at Columbia University. Neshich is the principal author of the STING suite of programs (with the current version being BlueStarSTING), and STING_DB. STING is a popular database and visualization tool providing the largest collection of physicochemical parameters that describe protein structure, stability, function, and interaction with other macromolecules. Neshich chaired a session at the meeting held in November 2004, where the Brazilian Association for Computational Biology and Bioinformatics (AB^3^C) was inaugurated. He was a member of the Board of Directors of the International Society for Computational Biology (ISCB) from 2003 to 2005 and chair of the Intelligent Systems in Molecular Biology (ISMB) 2006 conference in Fortaleza, Brazil.

## Genomics and Bioinformatics

In 1997 FAPESP began to invest both financial and human resources in genome sequencing. No central sequencing laboratory was created; rather, a network of laboratories was established. Genome assembly was performed on a single bioinformatics platform located in a laboratory at UNICAMP and placed under the leadership of two researchers who had just finished publishing the well-known book *Introduction to Computational Molecular Biology* [1]. A final result of this effort was the genetic map of Xillela fastidiosa published in *Nature* [2], a bacterium that attacks orange trees and costs this country more than $100 million a year. Soon after, the genomes of Xanthomonas citri, Xanthomonas campestris, Lifsonia xyli, and a strain of *Xillela* that attacks grapes in California were also deciphered (http://watson.fapesp.br/onsa/Genoma3.htm). All these bacteria cause considerable damage to Brazilian agriculture. Sugar cane, significant to the Brazilian economy, was the first plant to have its genome completely mapped out in Brazil. Brazil has established a very good record in genome sequencing by organizing the network of National laboratories to complete the eucalyptus genome and to participate, through an international network, in mapping out the banana, coffee, and rice genomes. Initiatives related to animal genomics have also sprouted bovine and pig genomes' sequencing initiated by Embrapa and the Network of laboratories of the southern Brazilian states, respectively. The latter is also deciphering the genome of the bacterium Mycoplasma hyopneumonia (http://www.brgene.lncc.br/), which causes pneumonia in pigs.

At the same time, human-health–related genome sequencing initiatives have been established, and the genome of Anopheles darlingi (the malaria mosquito) has been worked out with the participation of a number of laboratories distributed throughout Brazil. This national network was also set up to study a bacterium identified in the Amazon jungle region of Rio Negro, which produces an antibiotic substance possibly successful in treatment of the endemic Chagas disease. It has been postulated that this substance could be used to fight certain types of cancer, a project which is being explored in collaboration with the Ludwig Institute.

The list of organisms whose genomes are being sequenced in Brazil is still growing: in Rio de Janeiro state, the whole genome of bacterium Rhizobium tropici and *Bradyrhizobium Japonicum* which absorbs nitrogen from the air (and by doing so, improves the yield of the sugar cane and coffee crops) is currently being worked out by Embrapa and LNCC, as well as the genetic map of the fungus Crinipellis perniciosa (“witches' broom”) that causes drastically reduced production of cacao beans in Brazil (this genomic effort is coordinated by UNICAMP in Campinas, Sao Paulo). For a complete list of the genomics projects financed by the CNPq, see http://www.labinfo.lncc.br/index.php?option=com_content&task=view&id=18&itemid=130.

Independent from the above-described developments, Brazil has also invested in structural biology and built the first synchrotron in the Southern hemisphere in the 1990s (a second in Australia will begin operation this year), located again in São Paulo state in the city of Campinas, near UNICAMP. Since completion of the National Synchrotron Light Laboratory (LNLS) in July 1997, there has been a significant increase in the number of macromolecular structures deciphered in Brazil. Developments at the sequence and structure levels have fostered the need for related bioinformatics.

Brazil also found itself at the leading edge of cancer research by studying the genes of several of the locally most common malignant tumor diseases. This initiative was coordinated by the Ludwig Institute, and the project ended up making an important contribution to the map of the human genome.

Genomics, structural biology, bioinformatics, and computational biology have created an environment of integrated research. In Brazil, like elsewhere, many researchers with a broad spectrum of previous training and acquired skills, as well as trained bioinformaticians, have grasped these opportunities. In 2001 and 2004, the government offered financial support to research groups focused on bioinformatics applications, in support of genomics and structural biology. From approximately 30 projects nationwide approved by the CNPq in 2001, roughly half are from São Paulo state, recognizing the strength of the region.

## The Current State of Computational Biology: Services versus Research

Computational biology often involves both service (databases, software, consulting) and research. This is not different in Brazil. Funding agencies in Brazil must realize this as have agencies elsewhere. Another challenge is finding the right balance between large collaborative research projects and individual investigator-based research.

To any individual researcher, the large-scale projects offer more opportunities for service-oriented work and less opportunity for individual research. However, unlike what Sean Eddy describes in his “‘Antedisciplinary' Science” [3] as an analogy from the movie *Brazil*, I see that new, somewhat threatening, and a bit sterile environment more like the one from the Andrew Niccol's 1997 movie *Gattaca*. The idea seems to be effective that a researcher, over an extended time, devotes 40% of his time to service-oriented activities and 60% to fundamental research activities. This proportion is supported by Brazilian funding programs. If large-scale sequencing continues and an individual researcher's activity remains largely, if not exclusively, service to the larger team, this will not bode well for bioinformatics in Brazil.

## Is There Applied Science without Science To Be Applied?

Sir Roger Penrose, British physicist, recently said: “Today too much emphasis is given to what sometimes is referred to as ‘wealth creation,' or to what ‘research' is useful for.” Clearly, this is an important question that Brazil faces. The danger is that scientists are now being trained as “technology masters” and not as “knowledge decipherers.” In addition, some, if not many, research institutes are becoming technology institutes, a disturbing trend for the future. During the recent meeting of the Iberoamerican Network for Bioinformatics (http://rib.cecalc.ula.ve/) in Buenos Aires, a colleague and friend from Chile, David Holmes, while discussing the dilemma imposed by the pressure to be “useful” in science, pointed out that “there is no applied science without the science to apply.” The challenge would then seem to be to move from the basic characterization of the large amounts of sequence collected in our country to the full-scale analysis of these data. It is clear that we can quickly run out of the steam which is now available to genome projects and find ourselves in the uncharted area where the demand would be mainly for the expertise needed for interpreting data. The challenge here would be: how to interpret such data and which team should we be employing if, by now, all bioinformaticians, or at least a large number, have been trained to follow service orders and not so much to offer a creative response to data interpretation. The question that remains to be answered is: did genome projects actually catalyze development of bioinformatics in Brazil, or did they simply capture the available critical mass to perform a very important service? Clearly, one can argue that the role of bioinformaticians within the large genome sequencing projects is crucial for creatively solving some key biological problems. While this might be the case, there is no reason that such bioinformaticians should be (exclusively) just a part of a genome assembly project, rather than being an independent project leader or at least a researcher with a properly balanced ratio of activities in service and basic research (again, say 40:60). I would argue that in the latter case we would have much clearer opportunities for student training and the formation of “antedisciplinary” individuals. It appears that the current policies and project financing by the science funding agencies and research institutes in Brazil are yet to demonstrate that they are capable of taking care of this important problem. Interestingly, there is plenty of diversity in bioinformatics activities that can be identified in Brazilian academia today, in spite of a long period (almost a decade) with a predominant focus toward financing genomics-related bioinformatics activities. This seems to indicate that the ideal balance is not far from reach, nor was it jeopardized beyond repair in Brazil's “genomics” past.

## Knowledge Derived from Genomics Projects

According to Michael Galperin's report in the NAR Database issue from 2007 [4], there are 968 databases registered in this compendium, but only four claim the participation of Brazilian scientists, with three of them hosted within the .br domain. For years, from this geographic region, the only contributions to the list of almost 1,000 databases available worldwide was STING [5] and STING DB (http://sms.cbi.cnptia.embrapa.br/SMS/STINGm/SMSReport/)—a database of per-residue–reported descriptors (of protein sequences, structure, function, and stability), available for display both numerically and graphically, for either the public protein database (PDB) or local files. Recently, three new additions joined STING: MamMiBase (http://xavante.fmrp.usp.br/mammibase/)—for retrieving individual gene sequence alignments for genes in complete mammalian mitochondrial genomes, Tractor db—regulatory networks in gamma-proteobacteria database (http://www.tractor.lncc.br/), and ExInt (http://sege.ntu.edu.sg/wester/exint/)—a database that helps to identify common evolutionary patterns in higher eukaryotic genes for the study of intron loss/gain, sliding, splicing, retroposition, recombination, intron/exon duplication, etc. The first two are the products of joint effort by LNCC, UFRJ, and Fiocruz. The third includes participation of the Ludwig Institute from São Paulo, but the database is being hosted in Singapore. The large number of genomics efforts in Brazil seems to correlate poorly with the number of related/derived databases currently offered.

The number of genomics projects versus the number of databases seems to indicate that the bioinformatics community of Brazil has plenty of space to conquer, and that it is time to review the strategies used until now by the funding agencies and government policymakers.

## Brazilian Association for Bioinformatics and Computational Biology

Brazilian scientific societies need to show an interest in changing the current trend and to offer proper motivation to bioinformaticians who are keen to engage in thorough analysis of the information already gathered (and not necessarily locally, in geographic terms). The Brazilian Association for Bioinformatics and Computational Biology (AB^3^C) could be instrumental and should probably start exercising its influence by offering sound alternatives to policymakers, provided, of course, it can itself reach consensus on how to proceed.

The Brazilian Association for Bioinformatics and Computational Biology (AB^3^C: http://www.ab3c.org), an affiliated society of the International Society for Computational Biology (ISCB), was started in 2004. In October 2005 the first annual meeting of AB^3^C, the X-meeting, was held in Caxambu, in the Brazilian state of Minas Gerais (http://www.x-meeting.com). The accepted papers of that meeting were published in the open access journal *Genetics and Molecular Research* (http://www.funpecrp.com.br/gmr/year2004/vol4–3/index.htm). The X-meeting was a prelude to the second annual meeting of AB^3^C, which was held together with the Intelligent Systems for Molecular Biology (ISMB) 2006 conference in the city of Fortaleza in the state of Ceara, in Northeast Brazil. ISMB 2006 was an outstanding success for both ISCB and AB^3^C. The short and successful growth path of AB^3^C, currently with 227 members (2006 data), is a great overture for things to come; yet, we need to be very careful with regard to the responsibilities that this young Society needs to meet in the near future, specifically in terms of aiding governmental policymakers who map the directions for science in this country.

Also noteworthy is the Brazilian Bioinformatics Symposium (http://bsb2007.inf.puc-rio.br/) organized by the Brazilian Society for Computation—an additional forum for organizing and meeting for computational biologists in Brazil.

## Opportunities for Entrepreneurship

The Brazilian government encourages entrepreneurship related to biotechnology, and consequently computational biology. The already-established academic record and current activities are poised to catalyze new commercial activities which, if national and international venture capital is available, could impact the world market. However, government policies must be more decisive, especially in terms of supporting innovation and in changing the currently inefficient patent system aimed at general biotechnology, pharmaceutical, and software development areas. If Brazil is to accept that computational biology is the defining scientific endeavor of the twenty-first century, then the Brazilian government needs to work faster to open up new opportunities. Recent signals from the government are indeed positive. However, there is competition from countries such as India, China, and Russia, where the pool of skilled computational biologists is much larger and the publication record better established, at least according to ISI.

## Educational Activities

The future requires a broader pool of computational biologists. Brazil currently counts two University centers, USP and UFMG, which offer Ph.D. training in bioinformatics. Together with LNCC, which has an established program in training students for their M.Sc. in bioinformatics, these centers, and others that might establish programs, need to meet the demands of the future. Brazil looks to the current and future students to realize the promise of computational biology in Brazil. 
